# Supplementary material for: Thermal treatment and leaching of biochar alleviates plant growth inhibition from mobile organic compounds
Source: PeerJ. 2016 Aug 25;4:e2385. doi: 10.7717/peerj.2385 (PMC5012324; doi:10.7717/peerj.2385)
Supplement: Supplemental Information 1 — Supplementary Table S1. Simultaneous confidence intervals and test statistics for multiple comparisons (contrasts) of performance traits for ryegrass in experiment 1. Significant differences are in boldface type (p < 0.05). [file peerj-04-2385-s001.docx]

|  | Aboveground biomass (g) | | | |  | Belowground biomass (g) | | | |  | Leaf area (cm^2^) | | | |
| --- | --- | --- | --- | --- | --- | --- | --- | --- | --- | --- | --- | --- | --- | --- |
| Contrasts | Difference | Std.error | *t-value* | *P value* |  | Difference | Std.error | *t-value* | *P value* |  | Difference | Std.error | *t-value* | *P value* |
| 1. BC - Con | -44.02 | 17.18 | -3.61 | **0.006** |  | -4.53 | 1.05 | -4.32 | **<0.001** |  | -11.14 | 4.62 | -2.41 | 0.18 |
| 2. MB - Con | -39.30 | 13.07 | -3.01 | **0.04** |  | -4.59 | 1.13 | -4.07 | **0.001** |  | -10.31 | 4.97 | -2.08 | 0.34 |
| 3. SB - Con | -48.60 | 13.11 | -3.70 | **0.004** |  | -4.48 | 1.14 | -3.94 | **0.002** |  | -11.66 | 4.98 | -2.34 | 0.20 |
| 4. MFT - Con | -44.20 | 13.07 | -3.38 | **0.014** |  | -4.54 | 1.13 | -4.02 | **0.001** |  | -11.46 | 4.97 | -2.31 | 0.22 |
| 5. MB - SB | 9.27 | 8.32 | 1.11 | 0.93 |  | -0.104 | 0.7277 | -0.143 | 1.000 |  | 1.335 | 3.17 | 0.43 | 0.99 |
| 6. SB - MFT | -4.37 | 8.33 | -0.52 | 0.99 |  | 0.053 | 0.73 | 0.073 | 1.000 |  | -0.200 | 3.17 | -0.06 | 1.00 |
| 7. MFT - MB | -4.90 | 8.27 | -0.59 | 0.99 |  | 0.0512 | 0.71 | 0.072 | 1.000 |  | -1.15 | 3.14 | -0.37 | 1.00 |
| 8. BC (5) - Con | -31.03 | 13.50 | -2.29 | 0.28 |  | -4.43 | 1.16 | -3.80 | **0.003** |  | -5.88 | 5.13 | -1.15 | 0.92 |
| 9. BC (10) - Con | -41.70 | 13.50 | -3.09 | **0.032** |  | -4.14 | 1.16 | -3.56 | **0.008** |  | -10.30 | 5.13 | -2.01 | 0.39 |
| 10. BC (20) - Con | -43.23 | 13.50 | -3.20 | **0.023** |  | -4.16 | 1.16 | -3.57 | **0.007** |  | -10.75 | 5.13 | -2.01 | 0.33 |
| 11. BC (50) - Con | -60.12 | 13.57 | -4.43 | **<0.001** |  | -5.41 | 1.18 | -4.59 | **<0.001** |  | -17.63 | 5.15 | -3.42 | **0.01** |
| 12. BC (5) - (10) | 10.67 | 9.55 | 1.12 | 0.93 |  | -0.28 | 0.82 | -0.34 | 0.99 |  | 4.41 | 3.63 | 1.22 | 0.89 |
| 13. BC (5) - (20) | 12.20 | 9.54 | 1.28 | 0.86 |  | -0.265 | 0.823 | -0.322 | 0.97 |  | 4.48 | 3.63 | 1.34 | 0.83 |
| 14. BC (5) - (50) | 29.09 | 9.64 | 3.02 | **0.04** |  | 0.989 | 0.846 | 1.170 | 0.91 |  | 11.75 | 3.66 | 3.20 | **0.02** |
| 15. BC (10) - (20) | 1.53 | 9.55 | 0.161 | 1.00 |  | 0.017 | 0.823 | 0.020 | 1.00 |  | 0.45 | 3.63 | 0.12 | 1.00 |
| 16. BC (20) - (50) | 18.43 | 9.64 | 1.91 | **0.45** |  | 1.27 | 0.846 | 1.50 | 0.733 |  | 7.33 | 3.66 | 2.00 | 0.39 |
| 17. BC (20) - (50) | 16.90 | 9.65 | 1.75 | 0.56 |  | 1.25 | 0.846 | 1.48 | 0.746 |  | 6.89 | 3.66 | 1.88 | 0.47 |
| 18. Mixed - Con | -33.00 | 12.63 | -2.61 | 0.11 |  | -3.879 | 1.089 | -3.562 | **0.008** |  | -7.36 | 4.80 | -1.53 | 0.71 |
| 19. Top - Con | -55.05 | 12.65 | -4.35 | **<0.001** |  | -5.192 | 1.093 | -4.748 | **<0.001** |  | -14.93 | 4.80 | -3.10 | **0.03** |
| 20. Mixed - Top | 22.05 | 6.79 | 3.25 | **0.02** |  | 1.31 | 0.59 | 2.225 | 0.263 |  | 7.57 | 5.58 | 2.94 | **0.04** |
