# Supplementary material for: Thermal treatment and leaching of biochar alleviates plant growth inhibition from mobile organic compounds
Source: PeerJ. 2016 Aug 25;4:e2385. doi: 10.7717/peerj.2385 (PMC5012324; doi:10.7717/peerj.2385)
Supplement: Supplemental Information 2 — Supplementary Table S2. Simultaneous confidence intervals and test statistics for multiple comparisons (contrasts) of performance traits for clover in experiment 1. Significant differences are in boldface type (p < 0.05). [file peerj-04-2385-s002.docx]

|  | - Aboveground biomass (g) | | | |  | - Belowground biomass (g) | | | |  | - Leaf area (cm^2^) | | | |
| --- | --- | --- | --- | --- | --- | --- | --- | --- | --- | --- | --- | --- | --- | --- |
| - Contrasts | - Difference | - Std.error | - *t-value* | - *P value* |  | - Difference | - Std.error | - *t-value* | - *P value* |  | - Difference | - Std.error | - *t-value* | - *P value* |
| - 1. BC - Con | - 4.75 | - 6.22 | - 0.764 | - 0.99 |  | - 2.70 | - 3.67 | - 0.74 | - 0.99 |  | - -2.35 | - 2.08 | - -1.13 | - 0.92 |
| - 2. MB - Con | - 6.56 | - 6.65 | - 0.99 | - 0.96 |  | - 3.45 | - 3.92 | - 0.88 | - 0.98 |  | - -2.60 | - 2.24 | - -1.16 | - 0.91 |
| - 3. SB - Con | - 5.76 | - 6.63 | - 0.87 | - 0.98 |  | - 2.14 | - 3.91 | - 0.55 | - 0.99 |  | - -1.85 | - 2.23 | - -0.83 | - 0.99 |
| - 4. MFT - Con | - 1.93 | - 6.63 | - 0.29 | - 1.00 |  | - 2.51 | - 3.91 | - 0.64 | - 1.00 |  | - -2.60 | - 2.24 | - -1.15 | - 0.91 |
| - 5. MB - SB | - 0.80 | - 4.05 | - 0.20 | - 1.00 |  | - 1.31 | - 3.38 | - 0.55 | - 1.00 |  | - -0.75 | - 1.42 | - -0.53 | - 0.99 |
| - 6. SB - MFT | - 3.83 | - 4.02 | - 0.95 | - 0.97 |  | - -0.38 | - 2.39 | - -0.16 | - 1.00 |  | - 0.73 | - 1.42 | - 0.52 | - 0.99 |
| - 7. MFT - MB | - -4.63 | - 4.05 | - -1.14 | - 0.92 |  | - -0.94 | - 2.39 | - -0.40 | - 1.00 |  | - 0.02 | - 1.43 | - 0.01 | - 1.00 |
| - 8. BC (5) - Con | - 4.73 | - 6.83 | - 0.69 | - 0.99 |  | - 2.79 | - 4.03 | - 0.69 | - 0.99 |  | - -2.47 | - 2.33 | - -1.06 | - 0.94 |
| - 9. BC (10) - Con | - 5.23 | - 6.83 | - 0.77 | - 1.00 |  | - 2.72 | - 4.03 | - 0.68 | - 0.99 |  | - -0.32 | - 2.30 | - -0.14 | - 1.00 |
| - 10. BC (20) - Con | - 4.90 | - 6.87 | - 0.71 | - 1.00 |  | - 2.81 | - 4.05 | - 0.69 | - 0.99 |  | - -0.79 | - 2.30 | - -0.34 | - 1.00 |
| - 11. BC (50) - Con | - 4.13 | - 6.83 | - 0.61 | - 1.00 |  | - 2.49 | - 4.03 | - 0.62 | - 0.99 |  | - -5.82 | - 2.30 | - -2.52 | - 0.14 |
| - 12. BC (5) - (10) | - -0.50 | - 4.64 | - -0.108 | - 1.00 |  | - 0.07 | - 2.74 | - 0.024 | - 1.00 |  | - -2.15 | - 1.66 | - -1.29 | - 0.86 |
| - 13. BC (5) - (20) | - -0.17 | - 4.69 | - -0.04 | - 1.00 |  | - -0.02 | - 2.77 | - -0.01 | - 1.00 |  | - -1.70 | - 1.66 | - -1.00 | - 0.96 |
| - 14. BC (5) - (50) | - 0.60 | - 4.64 | - 0.13 | - 1.00 |  | - 0.30 | - 2.74 | - 0.11 | - 1.00 |  | - 3.35 | - 1.66 | - 2.01 | - 0.38 |
| - 15. BC (10) - (20) | - 0.33 | - 4.69 | - 0.07 | - 1.00 |  | - -0.08 | - 2.77 | - -0.03 | - 1.00 |  | - 0.47 | - 1.63 | - 0.29 | - 1.00 |
| - 16. BC (20) - (50) | - 1.10 | - 4.64 | - 0.24 | - 1.00 |  | - 0.23 | - 2.74 | - 0.09 | - 1.00 |  | - 5.50 | - 1.63 | - 3.37 | - **0.01** |
| - 17. BC (20) - (50) | - 0.77 | - 4.69 | - 0.16 | - 1.00 |  | - 0.32 | - 2.77 | - 0.11 | - 1.00 |  | - 5.03 | - 1.63 | - 3.08 | - **0.03** |
| - 18. Mixed - Con | - 2.40 | - 6.42 | - 0.37 | - 1.00 |  | - 2.49 | - 3.80 | - 0.66 | - 0.99 |  | - -3.04 | - 2.16 | - -1.40 | - 0.79 |
| - 19. Top - Con | - 7.10 | - 6.43 | - 1.10 | - 0.93 |  | - 2.91 | - 3.80 | - 0.77 | - 0.99 |  | - -1.66 | - 2.16 | - -0.77 | - 0.99 |
| - 20. Mixed - Top | - -4.70 | - 3.30 | - -1.42 | - 0.78 |  | - -0.43 | - 1.95 | - -0.21 | - 1.00 |  | - -1.37 | - 1.16 | - -1.18 | - 0.90 |
|  | - Nodules (# / root) | | |  |  |  |  |  |  |  |  |  |  |  |
| - 1. BC - Con | - 5.66 | - 2.49 | - 2.26 | - 0.24 |  |  |  |  |  |  |  |  |  |  |
| - 2. MB - Con | - 7.14 | - 2.67 | - 2.77 | - 0.76 |  |  |  |  |  |  |  |  |  |  |
| - 3. SB - Con | - 5.57 | - 2.66 | - 2.09 | - 0.33 |  |  |  |  |  |  |  |  |  |  |
| - 4. MFT - Con | - 3.98 | - 2.66 | - 1.50 | - 0.73 |  |  |  |  |  |  |  |  |  |  |
| - 5. MB - SB | - 1.84 | - 1.65 | - 1.12 | - 0.92 |  |  |  |  |  |  |  |  |  |  |
| - 6. SB - MFT | - 1.60 | - 1.61 | - 0.99 | - 0.96 |  |  |  |  |  |  |  |  |  |  |
| - 7. MFT - MB | - -3.43 | - 1.63 | - -2.09 | - 0.33 |  |  |  |  |  |  |  |  |  |  |
| - 8. BC (5) - Con | - 6.80 | - 2.75 | - 2.47 | - 0.15 |  |  |  |  |  |  |  |  |  |  |
| - 9. BC (10) - Con | - 7.31 | - 2.74 | - 3.03 | - 0.57 |  |  |  |  |  |  |  |  |  |  |
| - 10. BC (20) - Con | - 4.60 | - 2.75 | - 1.67 | - 0.61 |  |  |  |  |  |  |  |  |  |  |
| - 11. BC (50) - Con | - 2.91 | - 2.74 | - 1.06 | - 0.94 |  |  |  |  |  |  |  |  |  |  |
| - 12. BC (5) - (10) | - -1.50 | - 1.88 | - -0.80 | - 0.98 |  |  |  |  |  |  |  |  |  |  |
| - 13. BC (5) - (20) | - 2.20 | - 1.90 | - 1.15 | - 0.91 |  |  |  |  |  |  |  |  |  |  |
| - 14. BC (5) - (50) | - 3.89 | - 1.88 | - 2.06 | - 0.34 |  |  |  |  |  |  |  |  |  |  |
| - 15. BC (10) - (20) | - 3.70 | - 1.88 | - 1.97 | - 0.41 |  |  |  |  |  |  |  |  |  |  |
| - 16. BC (20) - (50) | - 5.40 | - 1.86 | - 2.89 | - 0.05 |  |  |  |  |  |  |  |  |  |  |
| - 17. BC (20) - (50) | - 1.70 | - 1.88 | - 0.89 | - 0.97 |  |  |  |  |  |  |  |  |  |  |
| - 18. Mixed - Con | - 5.09 | - 2.58 | - 1.97 | - 0.41 |  |  |  |  |  |  |  |  |  |  |
| - 19. Top - Con | - 6.22 | - 2.58 | - 2.41 | - 0.17 |  |  |  |  |  |  |  |  |  |  |
| - 20. Mixed - Top | - -1.13 | - 1.33 | - -0.85 | - 0.98 |  |  |  |  |  |  |  |  |  |  |
