# Supplementary material for: Thermal treatment and leaching of biochar alleviates plant growth inhibition from mobile organic compounds
Source: PeerJ. 2016 Aug 25;4:e2385. doi: 10.7717/peerj.2385 (PMC5012324; doi:10.7717/peerj.2385)
Supplement: Supplemental Information 3 — Supplementary Table S3. Simultaneous confidence intervals and test statistics for multiple comparisons (contrasts) of performance traits for ryegrass in experiment 2. Significant differences are in boldface type (p < 0.05). [file peerj-04-2385-s003.docx]

| **Contrasts** | aboveground biomass (g) | | | | | Belowground biomass (g) | | | | Leaf area (cm^2^) | | | | |  |
| --- | --- | --- | --- | --- | --- | --- | --- | --- | --- | --- | --- | --- | --- | --- | --- |
|  | Diff | Std. error | *t* | *p* | Diff | | Std. error | *t* | *p* | | Diff | Std. error | *t* | *p* | |
| 1. BC - Con | -45.11 | 14.43 | -3.13 | **0.03** | -30.97 | | 11.29 | -2.74 | 0.07 | | -15.95 | 3.64 | -4.38 | **<0.01** | |
| 2. BC (Mix) - Con | -43.21 | 17.40 | -2.48 | 0.13 | -32.49 | | 13.61 | -2.39 | 0.16 | | -13.86 | 4.40 | -3.15 | **0.02** | |
| 3. BC (Top) - Con | -47.00 | 16.75 | -2.81 | 0.06 | -29.45 | | 13.10 | -2.25 | 0.22 | | -18.04 | 4.23 | -4.26 | **<0.01** | |
| 4. BC (Top) - (Mix) | -3.79 | 18.28 | -0.21 | 1.00 | 3.04 | | 14.29 | 0.21 | 1.00 | | -4.17 | 4.62 | -0.90 | 0.96 | |
| 5. Treated - Con | -13.30 | 11.84 | -1.12 | 0.89 | -19.29 | | 9.26 | -2.08 | 0.30 | | -8.33 | 2.99 | -2.79 | 0.07 | |
| 6. Treated - BC | 31.81 | 9.95 | 3.20 | **0.02** | 11.68 | | 7.79 | 1.50 | 0.67 | | 7.62 | 2.51 | 3.03 | **0.03** | |
| 7. WW - BC | 29.23 | 11.07 | 2.64 | 0.09 | 10.99 | | 8.66 | 1.27 | 0.81 | | 6.35 | 2.80 | 2.27 | 0.21 | |
| 8. WW - Con | -15.88 | 12.79 | -1.24 | 0.83 | -19.98 | | 10.01 | -2.00 | 0.34 | | -9.60 | 3.23 | -2.97 | **0.04** | |
| 9. WW (24) - BC | 51.73 | 12.71 | 4.07 | **<0.01** | 25.08 | | 9.94 | 2.52 | 0.12 | | 11.40 | 3.21 | 3.55 | **<0.01** | |
| 10. WW (24) - Con | 6.63 | 14.23 | 0.47 | 1.00 | -5.89 | | 11.13 | -0.53 | 1.00 | | -4.55 | 3.60 | -1.27 | 0.82 | |
| 11. WW (0.5) - BC | 6.73 | 12.71 | 0.53 | 1.00 | -3.11 | | 9.94 | -0.31 | 1.00 | | 1.31 | 3.21 | 0.41 | 1.00 | |
| 12. WW (0.5) - Con | -38.38 | 14.23 | -2.70 | 0.08 | -34.08 | | 11.13 | -3.06 | **0.03** | | -14.64 | 3.60 | -4.07 | **<0.01** | |
| 13. Heat - Con | -11.58 | 12.27 | -0.94 | 0.95 | -18.83 | | 9.60 | -1.96 | 0.37 | | -7.49 | 3.10 | -2.42 | 0.15 | |
| 14. Heat - BC | 33.52 | 10.46 | 3.20 | **0.02** | 12.14 | | 8.18 | 1.48 | 0.68 | | 8.46 | 2.64 | 3.20 | **0.02** | |
| 15. Heat (50) - BC | 20.42 | 12.71 | 1.61 | 0.60 | 12.08 | | 9.94 | 1.22 | 0.84 | | 5.93 | 3.21 | 1.85 | 0.44 | |
| 16. Heat (50) - Con | -24.69 | 14.23 | -1.73 | 0.51 | -18.89 | | 11.13 | -1.70 | 0.54 | | -10.02 | 3.60 | -2.79 | 0.06 | |
| 17. Heat (100) - BC | 36.80 | 12.71 | 2.90 | **0.05** | 10.46 | | 9.94 | 1.05 | 0.91 | | 10.04 | 3.21 | 3.13 | **0.03** | |
| 18. Heat (100) - Con | -8.31 | 14.23 | -0.58 | 1.00 | -20.51 | | 11.13 | -1.84 | 0.44 | | -5.91 | 3.60 | -1.64 | 0.57 | |
| 19. Heat (150) - BC | 43.36 | 12.71 | 3.41 | **0.01** | 13.89 | | 9.94 | 1.40 | 0.74 | | 9.41 | 3.21 | 2.93 | **0.05** | |
| 20. Heat (150) - Con | -1.75 | 14.23 | -0.12 | 1.00 | -17.08 | | 11.13 | -1.53 | 0.65 | | -6.54 | 3.60 | -1.82 | 0.45 | |
| 21. Leach - Con | -66.44 | 14.23 | -4.67 | **<0.001** | -33.58 | | 11.13 | -3.02 | **0.02** | | -12.14 | 3.60 | -3.38 | **<0.01** | |
| 22. Leach - BC | -21.33 | 12.71 | -1.68 | 0.34 | -2.61 | | 9.94 | -0.26 | 1.00 | | 3.81 | 3.21 | 1.19 | 0.65 | |
| 23. Leach (24) - BC | -34.02 | 15.47 | -2.20 | 0.13 | -12.86 | | 12.10 | -1.06 | 0.73 | | -1.26 | 3.91 | -0.32 | 0.99 | |
| 24. Leach (24) - Con | -79.13 | 16.75 | -4.72 | **<0.001** | -43.83 | | 13.10 | -3.35 | **<0.01** | | -17.21 | 4.23 | -4.07 | **< 0.001** | |
| 25. Leach (0.5) - BC | 15.86 | 15.47 | 1.03 | 0.75 | 16.89 | | 12.10 | 1.40 | 0.51 | | 4.24 | 3.91 | 1.08 | 0.72 | |
| 26. Leach (0.5) - Con | -29.25 | 16.75 | -1.75 | 0.30 | -14.08 | | 13.10 | -1.07 | 0.72 | | -11.71 | 4.23 | -2.77 | **0.03** | |
