# Supplementary material for: Thermal treatment and leaching of biochar alleviates plant growth inhibition from mobile organic compounds
Source: PeerJ. 2016 Aug 25;4:e2385. doi: 10.7717/peerj.2385 (PMC5012324; doi:10.7717/peerj.2385)
Supplement: Supplemental Information 4 [file peerj-04-2385-s004.docx]

**Script for contrast tests used in experiments 1 and 2.**

* The script for the regression analysis in experiment 1 is available as a separate file.

Lolium. <- read.csv("~/Desktop/R scripts/Data for analyses/Lolium .csv")

Lolium.$ID <- factor (Lolium.$ID, levels = c ("MB5T", "MB5M", "MB10T", "MB10M","MB20T", "MB20M", "MB50T", "MB50M", "SB5T", "SB5M", "SB10T", "SB10M", "SB20T", "SB20M", "SB50T", "SB50M", "MFT5T", "MFT5M", "MFT10T", "MFT10M", "MFT20T", "MFT20M", "MFT50T", "MFT50M", "Con"))

## Lolium above ground biomass

Model1 <- lm(Abv~ID, data = Lolium.)

Contrats1 <- rbind (" 1. BC - Con" = c(1/24,1/24,1/24,1/24,1/24,1/24,1/24,1/24,1/24,1/24,1/24,1/24,1/24,1/24,1/24,1/24,1/24,1/24,1/24,1/24,1/24,1/24,1/24,1/24,-1),

" 2. MB - Con" = c(1/8,1/8,1/8,1/8,1/8,1/8,1/8,1/8,0,0,0,0,0,0,0,0,0,0,0,0,0,0,0,0,-1),

" 3. SB - Con" = c (0,0,0,0,0,0,0,0,1/8,1/8,1/8,1/8,1/8,1/8,1/8,1/8,0,0,0,0,0,0,0,0,-1),

" 4. MFT - Con"= c (0,0,0,0,0,0,0,0,0,0,0,0,0,0,0,0,1/8,1/8,1/8,1/8,1/8,1/8,1/8,1/8,1),

" 5. MB - SB" = c (1/8,1/8,1/8,1/8,1/8,1/8,1/8,1/8, -1/8, -1/8, -1/8, -1/8, -1/8, -1/8 , -1/8 , -1/8,0,0,0,0,0,0,0,0,0),

" 6. SB - MFT" = c (0,0,0,0,0,0,0,0,1/8,1/8,1/8,1/8,1/8,1/8,1/8,1/8, -1/8, -1/8, -1/8, -1/8, -1/8, -1/8 , -1/8 , -1/8, 0),

" 7. MFT - MB"= c (-1/8, -1/8, -1/8, -1/8, -1/8, -1/8 , -1/8 , -1/8,0,0,0,0,0,0,0,0,1/8,1/8,1/8,1/8,1/8,1/8,1/8,1/8,0),

" 8. b(5) - Con" = c(1/6,1/6,0,0,0,0,0,0,1/6,1/6,0,0,0,0,0,0,1/6,1/6,0,0,0,0,0,0,-1),

" 9. b(10) - Con"= c(0,0,1/6,1/6,0,0,0,0,0,0,1/6,1/6,0,0,0,0,0,0,1/6,1/6,0,0,0,0,-1),

" 10.b(20) - Con"= c (0,0,0,0,1/6,1/6,0,0,0,0,0,0,1/6,1/6,0,0,0,0,0,0,1/6,1/6,0,0,-1),

" 11.b(50)- Con" = c (0,0,0,0,0,0,1/6,1/6,0,0,0,0,0,0,1/6,1/6,0,0,0,0,0,0,1/6,1/6,-1),

" 12.b(5)-b(10)" = c (1/6,1/6,-1/6,-1/6,0,0,0,0,1/6,1/6,-1/6,-1/6,0,0,0,0,1/6,1/6,-1/6,-1/6,0,0,0,0,0),

" 13.b(5)-b(20)" = c (1/6,1/6,0,0,-1/6,-1/6,0,0,1/6,1/6,0,0,-1/6,-1/6,0,0,1/6,1/6,0,0,-1/6,-1/6,0,0,0),

" 14.b(5)-b(50)" = c (1/6,1/6,0,0,0,0,-1/6,-1/6,1/6,1/6,0,0,0,0,-1/6,-1/6,1/6,1/6,0,0,0,0,-1/6,-1/6,0),

" 15.b(10)-b(20)"= c (0,0,1/6,1/6,-1/6,-1/6,0,0,0,0,1/6,1/6,-1/6,-1/6,0,0,0,0,1/6,1/6,-1/6,-1/6,0,0,0),

" 16.b(10)-b(50)"= c (0,0,1/6,1/6,0,0,-1/6,-1/6,0,0,1/6,1/6,0,0,-1/6,-1/6,0,0,1/6,1/6,0,0,-1/6,-1/6,0),

" 17.b(20)-b(50)"= c (0,0,0,0,1/6,1/6,-1/6,-1/6,0,0,0,0,1/6,1/6,-1/6,-1/6,0,0,0,0,1/6,1/6,-1/6,-1/6,0),

" 18. Mix - Con" = c (0,1/12,0,1/12,0,1/12,0,1/12,0,1/12,0,1/12,0,1/12,0,1/12,0,1/12,0,1/12,0,1/12,0,1/12,-1),

" 19. Top - Con" = c (1/12,0,1/12,0,1/12,0,1/12,0,1/12,0,1/12,0,1/12,0,1/12,0,1/12,0,1/12,0,1/12,0,1/12,0,-1),

" 20. Mix - Top" = c (-1/12,1/12,-1/12,1/12,-1/12,1/12,-1/12,1/12,-1/12,1/12,-1/12,1/12,-1/12,1/12,-1/12,1/12,-1/12,1/12,-1/12,1/12,-1/12,1/12,-1/12,1/12,0))

comps1 <- glht(Model1, linfct = mcp (ID = Contrats1), alternative = "two.sided")

SC1 <- confint(comps1)

summary (SC1)

summary (comps1)

plot(comps1, main="", xlab = " Difference in Aboveground Biomass (g)")

par(mar=c (5,10,4,5)+.1)

x <- 1:8

plot (x,col = x)

## Lolium belowground

Model2 <- lm(Blgb~ID, data = Lolium.)

Contrats2 <- rbind (" 1. BC - Con" = c(1/24,1/24,1/24,1/24,1/24,1/24,1/24,1/24,1/24,1/24,1/24,1/24,1/24,1/24,1/24,1/24,1/24,1/24,1/24,1/24,1/24,1/24,1/24,1/24,-1),

" 2. MB - Con" = c(1/8,1/8,1/8,1/8,1/8,1/8,1/8,1/8,0,0,0,0,0,0,0,0,0,0,0,0,0,0,0,0,-1),

" 3. SB - Con" = c (0,0,0,0,0,0,0,0,1/8,1/8,1/8,1/8,1/8,1/8,1/8,1/8,0,0,0,0,0,0,0,0,-1),

" 4. MFT - Con"= c (0,0,0,0,0,0,0,0,0,0,0,0,0,0,0,0,1/8,1/8,1/8,1/8,1/8,1/8,1/8,1/8,-1),

" 5. MB - SB" = c (1/8,1/8,1/8,1/8,1/8,1/8,1/8,1/8, -1/8, -1/8, -1/8, -1/8, -1/8, -1/8 , -1/8 , -1/8,0,0,0,0,0,0,0,0,0),

" 6. SB - MFT" = c (0,0,0,0,0,0,0,0,1/8,1/8,1/8,1/8,1/8,1/8,1/8,1/8, -1/8, -1/8, -1/8, -1/8, -1/8, -1/8 , -1/8 , -1/8, 0),

" 7. MFT - MB"= c (-1/8, -1/8, -1/8, -1/8, -1/8, -1/8 , -1/8 , -1/8,0,0,0,0,0,0,0,0,1/8,1/8,1/8,1/8,1/8,1/8,1/8,1/8,0),

" 8. b(5) - Con" = c(1/6,1/6,0,0,0,0,0,0,1/6,1/6,0,0,0,0,0,0,1/6,1/6,0,0,0,0,0,0,-1),

" 9. b(10) - Con"= c(0,0,1/6,1/6,0,0,0,0,0,0,1/6,1/6,0,0,0,0,0,0,1/6,1/6,0,0,0,0,-1),

" 10.b(20) - Con"= c (0,0,0,0,1/6,1/6,0,0,0,0,0,0,1/6,1/6,0,0,0,0,0,0,1/6,1/6,0,0,-1),

" 11.b(50)- Con" = c (0,0,0,0,0,0,1/6,1/6,0,0,0,0,0,0,1/6,1/6,0,0,0,0,0,0,1/6,1/6,-1),

" 12.b(5)-b(10)" = c (1/6,1/6,-1/6,-1/6,0,0,0,0,1/6,1/6,-1/6,-1/6,0,0,0,0,1/6,1/6,-1/6,-1/6,0,0,0,0,0),

" 13.b(5)-b(20)" = c (1/6,1/6,0,0,-1/6,-1/6,0,0,1/6,1/6,0,0,-1/6,-1/6,0,0,1/6,1/6,0,0,-1/6,-1/6,0,0,0),

" 14.b(5)-b(50)" = c (1/6,1/6,0,0,0,0,-1/6,-1/6,1/6,1/6,0,0,0,0,-1/6,-1/6,1/6,1/6,0,0,0,0,-1/6,-1/6,0),

" 15.b(10)-b(20)"= c (0,0,1/6,1/6,-1/6,-1/6,0,0,0,0,1/6,1/6,-1/6,-1/6,0,0,0,0,1/6,1/6,-1/6,-1/6,0,0,0),

" 16.b(10)-b(50)"= c (0,0,1/6,1/6,0,0,-1/6,-1/6,0,0,1/6,1/6,0,0,-1/6,-1/6,0,0,1/6,1/6,0,0,-1/6,-1/6,0),

" 17.b(20)-b(50)"= c (0,0,0,0,1/6,1/6,-1/6,-1/6,0,0,0,0,1/6,1/6,-1/6,-1/6,0,0,0,0,1/6,1/6,-1/6,-1/6,0),

" 18. Mix - Con" = c (0,1/12,0,1/12,0,1/12,0,1/12,0,1/12,0,1/12,0,1/12,0,1/12,0,1/12,0,1/12,0,1/12,0,1/12,-1),

" 19. Top - Con" = c (1/12,0,1/12,0,1/12,0,1/12,0,1/12,0,1/12,0,1/12,0,1/12,0,1/12,0,1/12,0,1/12,0,1/12,0,-1),

" 20. Mix - Top" = c (-1/12,1/12,-1/12,1/12,-1/12,1/12,-1/12,1/12,-1/12,1/12,-1/12,1/12,-1/12,1/12,-1/12,1/12,-1/12,1/12,-1/12,1/12,-1/12,1/12,-1/12,1/12,0))

comps2 <- glht(Model2, linfct = mcp (ID = Contrats2), alternative = "two.sided")

SC2 <- confint(comps2)

summary (SC2)

summary (comps2)

plot(comps2, main="", xlab = " Difference in Belowground Biomass (g) ")

par(mar=c (5,7,4,5)+.1)

## Lolium Leaf area

Model3 <- lm(Larea~ID, data = Lolium.)

Contrats3 <- rbind (" 1. BC - Con" = c(1/24,1/24,1/24,1/24,1/24,1/24,1/24,1/24,1/24,1/24,1/24,1/24,1/24,1/24,1/24,1/24,1/24,1/24,1/24,1/24,1/24,1/24,1/24,1/24,-1),

" 2. MB - Con" = c(1/8,1/8,1/8,1/8,1/8,1/8,1/8,1/8,0,0,0,0,0,0,0,0,0,0,0,0,0,0,0,0,-1),

" 3. SB - Con" = c (0,0,0,0,0,0,0,0,1/8,1/8,1/8,1/8,1/8,1/8,1/8,1/8,0,0,0,0,0,0,0,0,-1),

" 4. MFT - Con"= c (0,0,0,0,0,0,0,0,0,0,0,0,0,0,0,0,1/8,1/8,1/8,1/8,1/8,1/8,1/8,1/8,-1),

" 5. MB - SB" = c (1/8,1/8,1/8,1/8,1/8,1/8,1/8,1/8, -1/8, -1/8, -1/8, -1/8, -1/8, -1/8 , -1/8 , -1/8,0,0,0,0,0,0,0,0,0),

" 6. SB - MFT" = c (0,0,0,0,0,0,0,0,1/8,1/8,1/8,1/8,1/8,1/8,1/8,1/8, -1/8, -1/8, -1/8, -1/8, -1/8, -1/8 , -1/8 , -1/8, 0),

" 7. MFT - MB"= c (-1/8, -1/8, -1/8, -1/8, -1/8, -1/8 , -1/8 , -1/8,0,0,0,0,0,0,0,0,1/8,1/8,1/8,1/8,1/8,1/8,1/8,1/8,0),

" 8. b(5) - Con" = c(1/6,1/6,0,0,0,0,0,0,1/6,1/6,0,0,0,0,0,0,1/6,1/6,0,0,0,0,0,0,-1),

" 9. b(10) - Con"= c(0,0,1/6,1/6,0,0,0,0,0,0,1/6,1/6,0,0,0,0,0,0,1/6,1/6,0,0,0,0,-1),

" 10.b(20) - Con"= c (0,0,0,0,1/6,1/6,0,0,0,0,0,0,1/6,1/6,0,0,0,0,0,0,1/6,1/6,0,0,-1),

" 11.b(50)- Con" = c (0,0,0,0,0,0,1/6,1/6,0,0,0,0,0,0,1/6,1/6,0,0,0,0,0,0,1/6,1/6,-1),

" 12.b(5)-b(10)" = c (1/6,1/6,-1/6,-1/6,0,0,0,0,1/6,1/6,-1/6,-1/6,0,0,0,0,1/6,1/6,-1/6,-1/6,0,0,0,0,0),

" 13.b(5)-b(20)" = c (1/6,1/6,0,0,-1/6,-1/6,0,0,1/6,1/6,0,0,-1/6,-1/6,0,0,1/6,1/6,0,0,-1/6,-1/6,0,0,0),

" 14.b(5)-b(50)" = c (1/6,1/6,0,0,0,0,-1/6,-1/6,1/6,1/6,0,0,0,0,-1/6,-1/6,1/6,1/6,0,0,0,0,-1/6,-1/6,0),

" 15.b(10)-b(20)"= c (0,0,1/6,1/6,-1/6,-1/6,0,0,0,0,1/6,1/6,-1/6,-1/6,0,0,0,0,1/6,1/6,-1/6,-1/6,0,0,0),

" 16.b(10)-b(50)"= c (0,0,1/6,1/6,0,0,-1/6,-1/6,0,0,1/6,1/6,0,0,-1/6,-1/6,0,0,1/6,1/6,0,0,-1/6,-1/6,0),

" 17.b(20)-b(50)"= c (0,0,0,0,1/6,1/6,-1/6,-1/6,0,0,0,0,1/6,1/6,-1/6,-1/6,0,0,0,0,1/6,1/6,-1/6,-1/6,0),

" 18. Mix - Con" = c (0,1/12,0,1/12,0,1/12,0,1/12,0,1/12,0,1/12,0,1/12,0,1/12,0,1/12,0,1/12,0,1/12,0,1/12,-1),

" 19. Top - Con" = c (1/12,0,1/12,0,1/12,0,1/12,0,1/12,0,1/12,0,1/12,0,1/12,0,1/12,0,1/12,0,1/12,0,1/12,0,-1),

" 20. Mix - Top" = c (-1/12,1/12,-1/12,1/12,-1/12,1/12,-1/12,1/12,-1/12,1/12,-1/12,1/12,-1/12,1/12,-1/12,1/12,-1/12,1/12,-1/12,1/12,-1/12,1/12,-1/12,1/12,0))

comps3 <- glht(Model3, linfct = mcp (ID = Contrats3), alternative = "two.sided")

SC3 <- confint(comps3)

summary (SC3)

summary (comps3)

plot(comps3, main="", xlab = " Difference in Leaf Area (cm^2) ")

par(mar=c (5,7,4,5)+.1)

## Lolium height

Model4 <- lm(height~ID, data = Lolium.)

Contrats4 <- rbind (" 1. BC - Con" = c(1/24,1/24,1/24,1/24,1/24,1/24,1/24,1/24,1/24,1/24,1/24,1/24,1/24,1/24,1/24,1/24,1/24,1/24,1/24,1/24,1/24,1/24,1/24,1/24,-1),

" 2. MB - Con" = c(1/8,1/8,1/8,1/8,1/8,1/8,1/8,1/8,0,0,0,0,0,0,0,0,0,0,0,0,0,0,0,0,-1),

" 3. SB - Con" = c (0,0,0,0,0,0,0,0,1/8,1/8,1/8,1/8,1/8,1/8,1/8,1/8,0,0,0,0,0,0,0,0,-1),

" 4. MFT - Con"= c (0,0,0,0,0,0,0,0,0,0,0,0,0,0,0,0,1/8,1/8,1/8,1/8,1/8,1/8,1/8,1/8,-1),

" 5. MB - SB" = c (1/8,1/8,1/8,1/8,1/8,1/8,1/8,1/8, -1/8, -1/8, -1/8, -1/8, -1/8, -1/8 , -1/8 , -1/8,0,0,0,0,0,0,0,0,0),

" 6. SB - MFT" = c (0,0,0,0,0,0,0,0,1/8,1/8,1/8,1/8,1/8,1/8,1/8,1/8, -1/8, -1/8, -1/8, -1/8, -1/8, -1/8 , -1/8 , -1/8, 0),

" 7. MFT - MB"= c (-1/8, -1/8, -1/8, -1/8, -1/8, -1/8 , -1/8 , -1/8,0,0,0,0,0,0,0,0,1/8,1/8,1/8,1/8,1/8,1/8,1/8,1/8,0),

" 8. b(5) - Con" = c(1/6,1/6,0,0,0,0,0,0,1/6,1/6,0,0,0,0,0,0,1/6,1/6,0,0,0,0,0,0,-1),

" 9. b(10) - Con"= c(0,0,1/6,1/6,0,0,0,0,0,0,1/6,1/6,0,0,0,0,0,0,1/6,1/6,0,0,0,0,-1),

" 10.b(20) - Con"= c (0,0,0,0,1/6,1/6,0,0,0,0,0,0,1/6,1/6,0,0,0,0,0,0,1/6,1/6,0,0,-1),

" 11.b(50)- Con" = c (0,0,0,0,0,0,1/6,1/6,0,0,0,0,0,0,1/6,1/6,0,0,0,0,0,0,1/6,1/6,-1),

" 12.b(5)-b(10)" = c (1/6,1/6,-1/6,-1/6,0,0,0,0,1/6,1/6,-1/6,-1/6,0,0,0,0,1/6,1/6,-1/6,-1/6,0,0,0,0,0),

" 13.b(5)-b(20)" = c (1/6,1/6,0,0,-1/6,-1/6,0,0,1/6,1/6,0,0,-1/6,-1/6,0,0,1/6,1/6,0,0,-1/6,-1/6,0,0,0),

" 14.b(5)-b(50)" = c (1/6,1/6,0,0,0,0,-1/6,-1/6,1/6,1/6,0,0,0,0,-1/6,-1/6,1/6,1/6,0,0,0,0,-1/6,-1/6,0),

" 15.b(10)-b(20)"= c (0,0,1/6,1/6,-1/6,-1/6,0,0,0,0,1/6,1/6,-1/6,-1/6,0,0,0,0,1/6,1/6,-1/6,-1/6,0,0,0),

" 16.b(10)-b(50)"= c (0,0,1/6,1/6,0,0,-1/6,-1/6,0,0,1/6,1/6,0,0,-1/6,-1/6,0,0,1/6,1/6,0,0,-1/6,-1/6,0),

" 17.b(20)-b(50)"= c (0,0,0,0,1/6,1/6,-1/6,-1/6,0,0,0,0,1/6,1/6,-1/6,-1/6,0,0,0,0,1/6,1/6,-1/6,-1/6,0),

" 18. Mix - Con" = c (0,1/12,0,1/12,0,1/12,0,1/12,0,1/12,0,1/12,0,1/12,0,1/12,0,1/12,0,1/12,0,1/12,0,1/12,-1),

" 19. Top - Con" = c (1/12,0,1/12,0,1/12,0,1/12,0,1/12,0,1/12,0,1/12,0,1/12,0,1/12,0,1/12,0,1/12,0,1/12,0,-1),

" 20. Mix - Top" = c (-1/12,1/12,-1/12,1/12,-1/12,1/12,-1/12,1/12,-1/12,1/12,-1/12,1/12,-1/12,1/12,-1/12,1/12,-1/12,1/12,-1/12,1/12,-1/12,1/12,-1/12,1/12,0))

comps4 <- glht(Model4, linfct = mcp (ID = Contrats4), alternative = "two.sided")

SC4 <- confint(comps4)

summary (SC4)

summary (comps4)

plot(SC4, main="", xlab = " Difference in Height (cm) ")

par(mar=c (5,7,4,5)+.1)

## Lolium pH

Model5 <- lm(pH~ID, data = Lolium.)

Contrats5 <- rbind (" 1. BC - Con" = c(1/24,1/24,1/24,1/24,1/24,1/24,1/24,1/24,1/24,1/24,1/24,1/24,1/24,1/24,1/24,1/24,1/24,1/24,1/24,1/24,1/24,1/24,1/24,1/24,-1),

" 2. MB - Con" = c(1/8,1/8,1/8,1/8,1/8,1/8,1/8,1/8,0,0,0,0,0,0,0,0,0,0,0,0,0,0,0,0,-1),

" 3. SB - Con" = c (0,0,0,0,0,0,0,0,1/8,1/8,1/8,1/8,1/8,1/8,1/8,1/8,0,0,0,0,0,0,0,0,-1),

" 4. MFT - Con"= c (0,0,0,0,0,0,0,0,0,0,0,0,0,0,0,0,1/8,1/8,1/8,1/8,1/8,1/8,1/8,1/8,-1),

" 5. MB - SB" = c (1/8,1/8,1/8,1/8,1/8,1/8,1/8,1/8, -1/8, -1/8, -1/8, -1/8, -1/8, -1/8 , -1/8 , -1/8,0,0,0,0,0,0,0,0,0),

" 6. SB - MFT" = c (0,0,0,0,0,0,0,0,1/8,1/8,1/8,1/8,1/8,1/8,1/8,1/8, -1/8, -1/8, -1/8, -1/8, -1/8, -1/8 , -1/8 , -1/8, 0),

" 7. MFT - MB"= c (-1/8, -1/8, -1/8, -1/8, -1/8, -1/8 , -1/8 , -1/8,0,0,0,0,0,0,0,0,1/8,1/8,1/8,1/8,1/8,1/8,1/8,1/8,0),

" 8. b(5) - Con" = c(1/6,1/6,0,0,0,0,0,0,1/6,1/6,0,0,0,0,0,0,1/6,1/6,0,0,0,0,0,0,-1),

" 9. b(10) - Con"= c(0,0,1/6,1/6,0,0,0,0,0,0,1/6,1/6,0,0,0,0,0,0,1/6,1/6,0,0,0,0,-1),

" 10.b(20) - Con"= c (0,0,0,0,1/6,1/6,0,0,0,0,0,0,1/6,1/6,0,0,0,0,0,0,1/6,1/6,0,0,-1),

" 11.b(50)- Con" = c (0,0,0,0,0,0,1/6,1/6,0,0,0,0,0,0,1/6,1/6,0,0,0,0,0,0,1/6,1/6,-1),

" 12.b(5)-b(10)" = c (1/6,1/6,-1/6,-1/6,0,0,0,0,1/6,1/6,-1/6,-1/6,0,0,0,0,1/6,1/6,-1/6,-1/6,0,0,0,0,0),

" 13.b(5)-b(20)" = c (1/6,1/6,0,0,-1/6,-1/6,0,0,1/6,1/6,0,0,-1/6,-1/6,0,0,1/6,1/6,0,0,-1/6,-1/6,0,0,0),

" 14.b(5)-b(50)" = c (1/6,1/6,0,0,0,0,-1/6,-1/6,1/6,1/6,0,0,0,0,-1/6,-1/6,1/6,1/6,0,0,0,0,-1/6,-1/6,0),

" 15.b(10)-b(20)"= c (0,0,1/6,1/6,-1/6,-1/6,0,0,0,0,1/6,1/6,-1/6,-1/6,0,0,0,0,1/6,1/6,-1/6,-1/6,0,0,0),

" 16.b(10)-b(50)"= c (0,0,1/6,1/6,0,0,-1/6,-1/6,0,0,1/6,1/6,0,0,-1/6,-1/6,0,0,1/6,1/6,0,0,-1/6,-1/6,0),

" 17.b(20)-b(50)"= c (0,0,0,0,1/6,1/6,-1/6,-1/6,0,0,0,0,1/6,1/6,-1/6,-1/6,0,0,0,0,1/6,1/6,-1/6,-1/6,0),

" 18. Mix - Con" = c (0,1/12,0,1/12,0,1/12,0,1/12,0,1/12,0,1/12,0,1/12,0,1/12,0,1/12,0,1/12,0,1/12,0,1/12,-1),

" 19. Top - Con" = c (1/12,0,1/12,0,1/12,0,1/12,0,1/12,0,1/12,0,1/12,0,1/12,0,1/12,0,1/12,0,1/12,0,1/12,0,-1),

" 20. Mix - Top" = c (-1/12,1/12,-1/12,1/12,-1/12,1/12,-1/12,1/12,-1/12,1/12,-1/12,1/12,-1/12,1/12,-1/12,1/12,-1/12,1/12,-1/12,1/12,-1/12,1/12,-1/12,1/12,0))

comps5 <- glht(Model5, linfct = mcp (ID = Contrats5), alternative = "two.sided")

SC5 <- confint(comps5)

summary (SC5)

summary (comps5)

plot(SC5, main="", xlab = " Difference in pH ")

par(mar=c (5,7,4,5)+.1)

##-------------------------------------------------------------------------------------------------------------------------

##-------------------------------------------------------------------------------------------------------------------------

trifolium <- read.csv("~/Desktop/R scripts/Data for analyses/trifolium.csv")

View(trifolium)

## Trifolium analyses

trifolium$ID <- factor (trifolium$ID, levels = c ("MB5T", "MB5M", "MB10T", "MB10M","MB20T", "MB20M", "MB50T", "MB50M", "SB5T", "SB5M", "SB10T", "SB10M", "SB20T", "SB20M", "SB50T", "SB50M", "MFT5T", "MFT5M", "MFT10T", "MFT10M", "MFT20T", "MFT20M", "MFT50T", "MFT50M", "Con"))

## Trifolium aboveground

Model6 <- lm(abvg~ID, data = trifolium)

Contrats6 <- rbind (" 1. BC - Con" = c(1/24,1/24,1/24,1/24,1/24,1/24,1/24,1/24,1/24,1/24,1/24,1/24,1/24,1/24,1/24,1/24,1/24,1/24,1/24,1/24,1/24,1/24,1/24,1/24,-1),

" 2. MB - Con" = c(1/8,1/8,1/8,1/8,1/8,1/8,1/8,1/8,0,0,0,0,0,0,0,0,0,0,0,0,0,0,0,0,-1),

" 3. SB - Con" = c (0,0,0,0,0,0,0,0,1/8,1/8,1/8,1/8,1/8,1/8,1/8,1/8,0,0,0,0,0,0,0,0,-1),

" 4. MFT - Con"= c (0,0,0,0,0,0,0,0,0,0,0,0,0,0,0,0,1/8,1/8,1/8,1/8,1/8,1/8,1/8,1/8,-1),

" 5. MB - SB" = c (1/8,1/8,1/8,1/8,1/8,1/8,1/8,1/8, -1/8, -1/8, -1/8, -1/8, -1/8, -1/8 , -1/8 , -1/8,0,0,0,0,0,0,0,0,0),

" 6. SB - MFT" = c (0,0,0,0,0,0,0,0,1/8,1/8,1/8,1/8,1/8,1/8,1/8,1/8, -1/8, -1/8, -1/8, -1/8, -1/8, -1/8 , -1/8 , -1/8, 0),

" 7. MFT - MB"= c (-1/8, -1/8, -1/8, -1/8, -1/8, -1/8 , -1/8 , -1/8,0,0,0,0,0,0,0,0,1/8,1/8,1/8,1/8,1/8,1/8,1/8,1/8,0),

" 8. b(5) - Con" = c(1/6,1/6,0,0,0,0,0,0,1/6,1/6,0,0,0,0,0,0,1/6,1/6,0,0,0,0,0,0,-1),

" 9. b(10) - Con"= c(0,0,1/6,1/6,0,0,0,0,0,0,1/6,1/6,0,0,0,0,0,0,1/6,1/6,0,0,0,0,-1),

" 10.b(20) - Con"= c (0,0,0,0,1/6,1/6,0,0,0,0,0,0,1/6,1/6,0,0,0,0,0,0,1/6,1/6,0,0,-1),

" 11.b(50)- Con" = c (0,0,0,0,0,0,1/6,1/6,0,0,0,0,0,0,1/6,1/6,0,0,0,0,0,0,1/6,1/6,-1),

" 12.b(5)-b(10)" = c (1/6,1/6,-1/6,-1/6,0,0,0,0,1/6,1/6,-1/6,-1/6,0,0,0,0,1/6,1/6,-1/6,-1/6,0,0,0,0,0),

" 13.b(5)-b(20)" = c (1/6,1/6,0,0,-1/6,-1/6,0,0,1/6,1/6,0,0,-1/6,-1/6,0,0,1/6,1/6,0,0,-1/6,-1/6,0,0,0),

" 14.b(5)-b(50)" = c (1/6,1/6,0,0,0,0,-1/6,-1/6,1/6,1/6,0,0,0,0,-1/6,-1/6,1/6,1/6,0,0,0,0,-1/6,-1/6,0),

" 15.b(10)-b(20)"= c (0,0,1/6,1/6,-1/6,-1/6,0,0,0,0,1/6,1/6,-1/6,-1/6,0,0,0,0,1/6,1/6,-1/6,-1/6,0,0,0),

" 16.b(10)-b(50)"= c (0,0,1/6,1/6,0,0,-1/6,-1/6,0,0,1/6,1/6,0,0,-1/6,-1/6,0,0,1/6,1/6,0,0,-1/6,-1/6,0),

" 17.b(20)-b(50)"= c (0,0,0,0,1/6,1/6,-1/6,-1/6,0,0,0,0,1/6,1/6,-1/6,-1/6,0,0,0,0,1/6,1/6,-1/6,-1/6,0),

" 18. Mix - Con" = c (0,1/12,0,1/12,0,1/12,0,1/12,0,1/12,0,1/12,0,1/12,0,1/12,0,1/12,0,1/12,0,1/12,0,1/12,-1),

" 19. Top - Con" = c (1/12,0,1/12,0,1/12,0,1/12,0,1/12,0,1/12,0,1/12,0,1/12,0,1/12,0,1/12,0,1/12,0,1/12,0,-1),

" 20. Mix - Top" = c (-1/12,1/12,-1/12,1/12,-1/12,1/12,-1/12,1/12,-1/12,1/12,-1/12,1/12,-1/12,1/12,-1/12,1/12,-1/12,1/12,-1/12,1/12,-1/12,1/12,-1/12,1/12,0))

comps6 <- glht(Model6, linfct = mcp (ID = Contrats6), alternative = "two.sided")

SC6 <- confint(comps6)

summary (SC6)

summary (comps6)

plot(comps6, main="", xlab = " Difference in Aboveground Biomass (g) ")

par(mar=c (5,7,4,5)+.1)

## Trifolium belowground

Model7 <- lm(blg~ID, data = trifolium)

Contrats7 <- rbind (" 1. BC - Con" = c(1/24,1/24,1/24,1/24,1/24,1/24,1/24,1/24,1/24,1/24,1/24,1/24,1/24,1/24,1/24,1/24,1/24,1/24,1/24,1/24,1/24,1/24,1/24,1/24,-1),

" 2. MB - Con" = c(1/8,1/8,1/8,1/8,1/8,1/8,1/8,1/8,0,0,0,0,0,0,0,0,0,0,0,0,0,0,0,0,-1),

" 3. SB - Con" = c (0,0,0,0,0,0,0,0,1/8,1/8,1/8,1/8,1/8,1/8,1/8,1/8,0,0,0,0,0,0,0,0,-1),

" 4. MFT - Con"= c (0,0,0,0,0,0,0,0,0,0,0,0,0,0,0,0,1/8,1/8,1/8,1/8,1/8,1/8,1/8,1/8,-1),

" 5. MB - SB" = c (1/8,1/8,1/8,1/8,1/8,1/8,1/8,1/8, -1/8, -1/8, -1/8, -1/8, -1/8, -1/8 , -1/8 , -1/8,0,0,0,0,0,0,0,0,0),

" 6. SB - MFT" = c (0,0,0,0,0,0,0,0,1/8,1/8,1/8,1/8,1/8,1/8,1/8,1/8, -1/8, -1/8, -1/8, -1/8, -1/8, -1/8 , -1/8 , -1/8, 0),

" 7. MFT - MB"= c (-1/8, -1/8, -1/8, -1/8, -1/8, -1/8 , -1/8 , -1/8,0,0,0,0,0,0,0,0,1/8,1/8,1/8,1/8,1/8,1/8,1/8,1/8,0),

" 8. b(5) - Con" = c(1/6,1/6,0,0,0,0,0,0,1/6,1/6,0,0,0,0,0,0,1/6,1/6,0,0,0,0,0,0,-1),

" 9. b(10) - Con"= c(0,0,1/6,1/6,0,0,0,0,0,0,1/6,1/6,0,0,0,0,0,0,1/6,1/6,0,0,0,0,-1),

" 10.b(20) - Con"= c (0,0,0,0,1/6,1/6,0,0,0,0,0,0,1/6,1/6,0,0,0,0,0,0,1/6,1/6,0,0,-1),

" 11.b(50)- Con" = c (0,0,0,0,0,0,1/6,1/6,0,0,0,0,0,0,1/6,1/6,0,0,0,0,0,0,1/6,1/6,-1),

" 12.b(5)-b(10)" = c (1/6,1/6,-1/6,-1/6,0,0,0,0,1/6,1/6,-1/6,-1/6,0,0,0,0,1/6,1/6,-1/6,-1/6,0,0,0,0,0),

" 13.b(5)-b(20)" = c (1/6,1/6,0,0,-1/6,-1/6,0,0,1/6,1/6,0,0,-1/6,-1/6,0,0,1/6,1/6,0,0,-1/6,-1/6,0,0,0),

" 14.b(5)-b(50)" = c (1/6,1/6,0,0,0,0,-1/6,-1/6,1/6,1/6,0,0,0,0,-1/6,-1/6,1/6,1/6,0,0,0,0,-1/6,-1/6,0),

" 15.b(10)-b(20)"= c (0,0,1/6,1/6,-1/6,-1/6,0,0,0,0,1/6,1/6,-1/6,-1/6,0,0,0,0,1/6,1/6,-1/6,-1/6,0,0,0),

" 16.b(10)-b(50)"= c (0,0,1/6,1/6,0,0,-1/6,-1/6,0,0,1/6,1/6,0,0,-1/6,-1/6,0,0,1/6,1/6,0,0,-1/6,-1/6,0),

" 17.b(20)-b(50)"= c (0,0,0,0,1/6,1/6,-1/6,-1/6,0,0,0,0,1/6,1/6,-1/6,-1/6,0,0,0,0,1/6,1/6,-1/6,-1/6,0),

" 18. Mix - Con" = c (0,1/12,0,1/12,0,1/12,0,1/12,0,1/12,0,1/12,0,1/12,0,1/12,0,1/12,0,1/12,0,1/12,0,1/12,-1),

" 19. Top - Con" = c (1/12,0,1/12,0,1/12,0,1/12,0,1/12,0,1/12,0,1/12,0,1/12,0,1/12,0,1/12,0,1/12,0,1/12,0,-1),

" 20. Mix - Top" = c (-1/12,1/12,-1/12,1/12,-1/12,1/12,-1/12,1/12,-1/12,1/12,-1/12,1/12,-1/12,1/12,-1/12,1/12,-1/12,1/12,-1/12,1/12,-1/12,1/12,-1/12,1/12,0))

comps7 <- glht(Model7, linfct = mcp (ID = Contrats7), alternative = "two.sided")

SC7 <- confint(comps7)

summary (SC7)

summary (comps7)

plot(comps7, main="", xlab = " Difference in Belowground Biomass (g) ")

par(mar=c (5,7,4,5)+.1)

## Trifolium Leaf area

Model8 <- lm(Larea~ID, data = trifolium)

Contrats8 <- rbind (" 1. BC - Con" = c(1/24,1/24,1/24,1/24,1/24,1/24,1/24,1/24,1/24,1/24,1/24,1/24,1/24,1/24,1/24,1/24,1/24,1/24,1/24,1/24,1/24,1/24,1/24,1/24,-1),

" 2. MB - Con" = c(1/8,1/8,1/8,1/8,1/8,1/8,1/8,1/8,0,0,0,0,0,0,0,0,0,0,0,0,0,0,0,0,-1),

" 3. SB - Con" = c (0,0,0,0,0,0,0,0,1/8,1/8,1/8,1/8,1/8,1/8,1/8,1/8,0,0,0,0,0,0,0,0,-1),

" 4. MFT - Con"= c (0,0,0,0,0,0,0,0,0,0,0,0,0,0,0,0,1/8,1/8,1/8,1/8,1/8,1/8,1/8,1/8,-1),

" 5. MB - SB" = c (1/8,1/8,1/8,1/8,1/8,1/8,1/8,1/8, -1/8, -1/8, -1/8, -1/8, -1/8, -1/8 , -1/8 , -1/8,0,0,0,0,0,0,0,0,0),

" 6. SB - MFT" = c (0,0,0,0,0,0,0,0,1/8,1/8,1/8,1/8,1/8,1/8,1/8,1/8, -1/8, -1/8, -1/8, -1/8, -1/8, -1/8 , -1/8 , -1/8, 0),

" 7. MFT - MB"= c (-1/8, -1/8, -1/8, -1/8, -1/8, -1/8 , -1/8 , -1/8,0,0,0,0,0,0,0,0,1/8,1/8,1/8,1/8,1/8,1/8,1/8,1/8,0),

" 8. b(5) - Con" = c(1/6,1/6,0,0,0,0,0,0,1/6,1/6,0,0,0,0,0,0,1/6,1/6,0,0,0,0,0,0,-1),

" 9. b(10) - Con"= c(0,0,1/6,1/6,0,0,0,0,0,0,1/6,1/6,0,0,0,0,0,0,1/6,1/6,0,0,0,0,-1),

" 10.b(20) - Con"= c (0,0,0,0,1/6,1/6,0,0,0,0,0,0,1/6,1/6,0,0,0,0,0,0,1/6,1/6,0,0,-1),

" 11.b(50)- Con" = c (0,0,0,0,0,0,1/6,1/6,0,0,0,0,0,0,1/6,1/6,0,0,0,0,0,0,1/6,1/6,-1),

" 12.b(5)-b(10)" = c (1/6,1/6,-1/6,-1/6,0,0,0,0,1/6,1/6,-1/6,-1/6,0,0,0,0,1/6,1/6,-1/6,-1/6,0,0,0,0,0),

" 13.b(5)-b(20)" = c (1/6,1/6,0,0,-1/6,-1/6,0,0,1/6,1/6,0,0,-1/6,-1/6,0,0,1/6,1/6,0,0,-1/6,-1/6,0,0,0),

" 14.b(5)-b(50)" = c (1/6,1/6,0,0,0,0,-1/6,-1/6,1/6,1/6,0,0,0,0,-1/6,-1/6,1/6,1/6,0,0,0,0,-1/6,-1/6,0),

" 15.b(10)-b(20)"= c (0,0,1/6,1/6,-1/6,-1/6,0,0,0,0,1/6,1/6,-1/6,-1/6,0,0,0,0,1/6,1/6,-1/6,-1/6,0,0,0),

" 16.b(10)-b(50)"= c (0,0,1/6,1/6,0,0,-1/6,-1/6,0,0,1/6,1/6,0,0,-1/6,-1/6,0,0,1/6,1/6,0,0,-1/6,-1/6,0),

" 17.b(20)-b(50)"= c (0,0,0,0,1/6,1/6,-1/6,-1/6,0,0,0,0,1/6,1/6,-1/6,-1/6,0,0,0,0,1/6,1/6,-1/6,-1/6,0),

" 18. Mix - Con" = c (0,1/12,0,1/12,0,1/12,0,1/12,0,1/12,0,1/12,0,1/12,0,1/12,0,1/12,0,1/12,0,1/12,0,1/12,-1),

" 19. Top - Con" = c (1/12,0,1/12,0,1/12,0,1/12,0,1/12,0,1/12,0,1/12,0,1/12,0,1/12,0,1/12,0,1/12,0,1/12,0,-1),

" 20. Mix - Top" = c (-1/12,1/12,-1/12,1/12,-1/12,1/12,-1/12,1/12,-1/12,1/12,-1/12,1/12,-1/12,1/12,-1/12,1/12,-1/12,1/12,-1/12,1/12,-1/12,1/12,-1/12,1/12,0))

comps8 <- glht(Model8, linfct = mcp (ID = Contrats8), alternative = "two.sided")

SC8 <- confint(comps8)

summary (SC8)

summary (comps8)

plot(comps8, main="", xlab = " Difference in Leaf Area (cm^2) ")

par(mar=c (5,7,4,5)+.1)

## Trifolium nodules

Model9 <- lm(nods~ID, data = trifolium)

Contrats9 <- rbind(

" 1. BC - Con" = c(1/24,1/24,1/24,1/24,1/24,1/24,1/24,1/24,1/24,1/24,1/24,1/24,1/24,1/24,1/24,1/24,1/24,1/24,1/24,1/24,1/24,1/24,1/24,1/24,-1),

" 2. MB - Con" = c(1/8,1/8,1/8,1/8,1/8,1/8,1/8,1/8,0,0,0,0,0,0,0,0,0,0,0,0,0,0,0,0,-1),

" 3. SB - Con" = c (0,0,0,0,0,0,0,0,1/8,1/8,1/8,1/8,1/8,1/8,1/8,1/8,0,0,0,0,0,0,0,0,-1),

" 4. MFT - Con"= c (0,0,0,0,0,0,0,0,0,0,0,0,0,0,0,0,1/8,1/8,1/8,1/8,1/8,1/8,1/8,1/8,-1),

" 5. MB - SB" = c (1/8,1/8,1/8,1/8,1/8,1/8,1/8,1/8, -1/8, -1/8, -1/8, -1/8, -1/8, -1/8 , -1/8 , -1/8,0,0,0,0,0,0,0,0,0),

" 6. SB - MFT" = c (0,0,0,0,0,0,0,0,1/8,1/8,1/8,1/8,1/8,1/8,1/8,1/8, -1/8, -1/8, -1/8, -1/8, -1/8, -1/8 , -1/8 , -1/8, 0),

" 7. MFT - MB"= c (-1/8, -1/8, -1/8, -1/8, -1/8, -1/8 , -1/8 , -1/8,0,0,0,0,0,0,0,0,1/8,1/8,1/8,1/8,1/8,1/8,1/8,1/8,0),

" 8. b(5) - Con" = c(1/6,1/6,0,0,0,0,0,0,1/6,1/6,0,0,0,0,0,0,1/6,1/6,0,0,0,0,0,0,-1),

" 9. b(10) - Con"= c(0,0,1/6,1/6,0,0,0,0,0,0,1/6,1/6,0,0,0,0,0,0,1/6,1/6,0,0,0,0,-1),

" 10.b(20) - Con"= c (0,0,0,0,1/6,1/6,0,0,0,0,0,0,1/6,1/6,0,0,0,0,0,0,1/6,1/6,0,0,-1),

" 11.b(50)- Con" = c (0,0,0,0,0,0,1/6,1/6,0,0,0,0,0,0,1/6,1/6,0,0,0,0,0,0,1/6,1/6,-1),

" 12.b(5)-b(10)" = c (1/6,1/6,-1/6,-1/6,0,0,0,0,1/6,1/6,-1/6,-1/6,0,0,0,0,1/6,1/6,-1/6,-1/6,0,0,0,0,0),

" 13.b(5)-b(20)" = c (1/6,1/6,0,0,-1/6,-1/6,0,0,1/6,1/6,0,0,-1/6,-1/6,0,0,1/6,1/6,0,0,-1/6,-1/6,0,0,0),

" 14.b(5)-b(50)" = c (1/6,1/6,0,0,0,0,-1/6,-1/6,1/6,1/6,0,0,0,0,-1/6,-1/6,1/6,1/6,0,0,0,0,-1/6,-1/6,0),

" 15.b(10)-b(20)"= c (0,0,1/6,1/6,-1/6,-1/6,0,0,0,0,1/6,1/6,-1/6,-1/6,0,0,0,0,1/6,1/6,-1/6,-1/6,0,0,0),

" 16.b(10)-b(50)"= c (0,0,1/6,1/6,0,0,-1/6,-1/6,0,0,1/6,1/6,0,0,-1/6,-1/6,0,0,1/6,1/6,0,0,-1/6,-1/6,0),

" 17.b(20)-b(50)"= c (0,0,0,0,1/6,1/6,-1/6,-1/6,0,0,0,0,1/6,1/6,-1/6,-1/6,0,0,0,0,1/6,1/6,-1/6,-1/6,0),

" 18. Mix - Con" = c (0,1/12,0,1/12,0,1/12,0,1/12,0,1/12,0,1/12,0,1/12,0,1/12,0,1/12,0,1/12,0,1/12,0,1/12,-1),

" 19. Top - Con" = c (1/12,0,1/12,0,1/12,0,1/12,0,1/12,0,1/12,0,1/12,0,1/12,0,1/12,0,1/12,0,1/12,0,1/12,0,-1),

" 20. Mix - Top" = c (-1/12,1/12,-1/12,1/12,-1/12,1/12,-1/12,1/12,-1/12,1/12,-1/12,1/12,-1/12,1/12,-1/12,1/12,-1/12,1/12,-1/12,1/12,-1/12,1/12,-1/12,1/12,0))

comps9 <- glht(Model9, linfct = mcp (ID = Contrats9), alternative = "two.sided")

SC9 <- confint(comps9)

summary (SC9)

summary (comps9)

plot(SC9, main="", xlab = " Difference in nodules ")

par(mar=c (5,7,4,5)+.1)

## Trifolium pH

Model10 <- lm(pH~ID, data = trifolium)

Contrats10 <- rbind (" 1. BC - Con" = c(1/24,1/24,1/24,1/24,1/24,1/24,1/24,1/24,1/24,1/24,1/24,1/24,1/24,1/24,1/24,1/24,1/24,1/24,1/24,1/24,1/24,1/24,1/24,1/24,-1),

" 2. MB - Con" = c(1/8,1/8,1/8,1/8,1/8,1/8,1/8,1/8,0,0,0,0,0,0,0,0,0,0,0,0,0,0,0,0,-1),

" 3. SB - Con" = c (0,0,0,0,0,0,0,0,1/8,1/8,1/8,1/8,1/8,1/8,1/8,1/8,0,0,0,0,0,0,0,0,-1),

" 4. MFT - Con"= c (0,0,0,0,0,0,0,0,0,0,0,0,0,0,0,0,1/8,1/8,1/8,1/8,1/8,1/8,1/8,1/8,-1),

" 5. MB - SB" = c (1/8,1/8,1/8,1/8,1/8,1/8,1/8,1/8, -1/8, -1/8, -1/8, -1/8, -1/8, -1/8 , -1/8 , -1/8,0,0,0,0,0,0,0,0,0),

" 6. SB - MFT" = c (0,0,0,0,0,0,0,0,1/8,1/8,1/8,1/8,1/8,1/8,1/8,1/8, -1/8, -1/8, -1/8, -1/8, -1/8, -1/8 , -1/8 , -1/8, 0),

" 7. MFT - MB"= c (-1/8, -1/8, -1/8, -1/8, -1/8, -1/8 , -1/8 , -1/8,0,0,0,0,0,0,0,0,1/8,1/8,1/8,1/8,1/8,1/8,1/8,1/8,0),

" 8. b(5) - Con" = c(1/6,1/6,0,0,0,0,0,0,1/6,1/6,0,0,0,0,0,0,1/6,1/6,0,0,0,0,0,0,-1),

" 9. b(10) - Con"= c(0,0,1/6,1/6,0,0,0,0,0,0,1/6,1/6,0,0,0,0,0,0,1/6,1/6,0,0,0,0,-1),

" 10.b(20) - Con"= c (0,0,0,0,1/6,1/6,0,0,0,0,0,0,1/6,1/6,0,0,0,0,0,0,1/6,1/6,0,0,-1),

" 11.b(50)- Con" = c (0,0,0,0,0,0,1/6,1/6,0,0,0,0,0,0,1/6,1/6,0,0,0,0,0,0,1/6,1/6,-1),

" 12.b(5)-b(10)" = c (1/6,1/6,-1/6,-1/6,0,0,0,0,1/6,1/6,-1/6,-1/6,0,0,0,0,1/6,1/6,-1/6,-1/6,0,0,0,0,0),

" 13.b(5)-b(20)" = c (1/6,1/6,0,0,-1/6,-1/6,0,0,1/6,1/6,0,0,-1/6,-1/6,0,0,1/6,1/6,0,0,-1/6,-1/6,0,0,0),

" 14.b(5)-b(50)" = c (1/6,1/6,0,0,0,0,-1/6,-1/6,1/6,1/6,0,0,0,0,-1/6,-1/6,1/6,1/6,0,0,0,0,-1/6,-1/6,0),

" 15.b(10)-b(20)"= c (0,0,1/6,1/6,-1/6,-1/6,0,0,0,0,1/6,1/6,-1/6,-1/6,0,0,0,0,1/6,1/6,-1/6,-1/6,0,0,0),

" 16.b(10)-b(50)"= c (0,0,1/6,1/6,0,0,-1/6,-1/6,0,0,1/6,1/6,0,0,-1/6,-1/6,0,0,1/6,1/6,0,0,-1/6,-1/6,0),

" 17.b(20)-b(50)"= c (0,0,0,0,1/6,1/6,-1/6,-1/6,0,0,0,0,1/6,1/6,-1/6,-1/6,0,0,0,0,1/6,1/6,-1/6,-1/6,0),

" 18. Mix - Con" = c (0,1/12,0,1/12,0,1/12,0,1/12,0,1/12,0,1/12,0,1/12,0,1/12,0,1/12,0,1/12,0,1/12,0,1/12,-1),

" 19. Top - Con" = c (1/12,0,1/12,0,1/12,0,1/12,0,1/12,0,1/12,0,1/12,0,1/12,0,1/12,0,1/12,0,1/12,0,1/12,0,-1),

" 20. Mix - Top" = c (-1/12,1/12,-1/12,1/12,-1/12,1/12,-1/12,1/12,-1/12,1/12,-1/12,1/12,-1/12,1/12,-1/12,1/12,-1/12,1/12,-1/12,1/12,-1/12,1/12,-1/12,1/12,0))

comps10 <- glht(Model10, linfct = mcp (ID = Contrats10), alternative = "two.sided")

SC10 <- confint(comps10)

summary (SC10)

summary (comps10)

plot(SC10, main="", xlab = " Difference in pH ")

par(mar=c (5,7,4,5)+.1)

##-------------------------------------------------------------------------------------------------------------------------------------------------------------------------------------------

##-------------------------------------------------------------------------------------------------------------------------------------------------------------------------------------------

Washing <- read.csv("~/Desktop/R scripts/Data for analyses/Washing.csv")

View(Washing)

Washing$ID <- factor (Washing$ID, levels = c ("WW24T", "WW24M", "WW.5T", "WW.5M", "EF24", "EF.5", "H50T", "H50M", "H100T", "H100M", "H150T", "H150M", "BCM", "BCT", "Con"))

Model11 <- lm(abv~ID, data = Washing)

Contrats11 <- rbind ("1. BC - Con" = c (0,0,0,0,0,0,0,0,0,0,0,0,1/2,1/2,-1),

"2. BCM - Con" = c(0,0,0,0,0,0,0,0,0,0,0,0,1,0,-1),

"3. BCT - Con" = c(0,0,0,0,0,0,0,0,0,0,0,0,0,1,-1),

"4. BCT - BCM" = c(0,0,0,0,0,0,0,0,0,0,0,0,-1,1,0),

"5. Trt - Con" = c(1/10,1/10,1/10,1/10,0,0,1/10,1/10,1/10,1/10,1/10,1/10,0,0,-1),

"6. Trt - BC" = c(1/10,1/10,1/10,1/10,0,0,1/10,1/10,1/10,1/10,1/10,1/10,-1/2,-1/2,0),

"7. WW - BC" = c (1/4,1/4,1/4,1/4,0,0,0,0,0,0,0,0,-1/2,-1/2,0),

"8. WW - Con" = c(1/4,1/4,1/4,1/4,0,0,0,0,0,0,0,0,0,0,-1),

"9. WW24 - BC" = c(1/2,1/2,0,0,0,0,0,0,0,0,0,0,-1/2,-1/2,0),

"10. WW24 - Con" = c(1/2,1/2,0,0,0,0,0,0,0,0,0,0,0,0,-1),

"11. WW.5 - BC" = c(0,0,1/2,1/2,0,0,0,0,0,0,0,0,-1/2,-1/2,0),

"12. WW.5 - Con"= c(0,0,1/2,1/2,0,0,0,0,0,0,0,0,0,0,-1),

"13. H - Con" = c (0,0,0,0,0,0,1/6,1/6,1/6,1/6,1/6,1/6,0,0,-1),

"14. H - BC" = c (0,0,0,0,0,0,1/6,1/6,1/6,1/6,1/6,1/6,-1/2,-1/2,0),

"15. H50 - BC" = c(0,0,0,0,0,0,1/2,1/2,0,0,0,0,-1/2,-1/2,0),

"16. H50 - Con"= c(0,0,0,0,0,0,1/2,1/2,0,0,0,0,0,0,-1),

"17. H100 - BC" = c(0,0,0,0,0,0,0,0,1/2,1/2,0,0,-1/2,-1/2,0),

"18. H100 - Con" = c(0,0,0,0,0,0,0,0,1/2,1/2,0,0,0,0,-1),

"19. H150 - BC" = c(0,0,0,0,0,0,0,0,0,0,1/2,1/2,-1/2,-1/2,0),

"20. H150 - Con" = c(0,0,0,0,0,0,0,0,0,0,1/2,1/2,0,0,-1))

comps11 <- glht(Model11, linfct = mcp (ID = Contrats11), alternative = "two.sided")

SC11 <- confint(comps11)

summary (SC11)

View(SC11)

plot(SC11, main="", xlab = " Difference in Above Ground Biomass (g) ")

par(mar=c (5,10,4,5)+.1)

## Washing belowground

Model12 <- lm(blg~ID, data = Washing)

Contrats12 <- rbind ("1. BC - Con" = c (0,0,0,0,0,0,0,0,0,0,0,0,1/2,1/2,-1),

"2. BCM - Con" = c(0,0,0,0,0,0,0,0,0,0,0,0,1,0,-1),

"3. BCT - Con" = c(0,0,0,0,0,0,0,0,0,0,0,0,0,1,-1),

"4. BCT - BCM" = c(0,0,0,0,0,0,0,0,0,0,0,0,-1,1,0),

"5. Trt - Con" = c(1/10,1/10,1/10,1/10,0,0,1/10,1/10,1/10,1/10,1/10,1/10,0,0,-1),

"6. Trt - BC" = c(1/10,1/10,1/10,1/10,0,0,1/10,1/10,1/10,1/10,1/10,1/10,-1/2,-1/2,0),

"7. WW - BC" = c (1/4,1/4,1/4,1/4,0,0,0,0,0,0,0,0,-1/2,-1/2,0),

"8. WW - Con" = c(1/4,1/4,1/4,1/4,0,0,0,0,0,0,0,0,0,0,-1),

"9. WW24 - BC" = c(1/2,1/2,0,0,0,0,0,0,0,0,0,0,-1/2,-1/2,0),

"10. WW24 - Con" = c(1/2,1/2,0,0,0,0,0,0,0,0,0,0,0,0,-1),

"11. WW.5 - BC" = c(0,0,1/2,1/2,0,0,0,0,0,0,0,0,-1/2,-1/2,0),

"12. WW.5 - Con"= c(0,0,1/2,1/2,0,0,0,0,0,0,0,0,0,0,-1),

"13. H - Con" = c (0,0,0,0,0,0,1/6,1/6,1/6,1/6,1/6,1/6,0,0,-1),

"14. H - BC" = c (0,0,0,0,0,0,1/6,1/6,1/6,1/6,1/6,1/6,-1/2,-1/2,0),

"15. H50 - BC" = c(0,0,0,0,0,0,1/2,1/2,0,0,0,0,-1/2,-1/2,0),

"16. H50 - Con"= c(0,0,0,0,0,0,1/2,1/2,0,0,0,0,0,0,-1),

"17. H100 - BC" = c(0,0,0,0,0,0,0,0,1/2,1/2,0,0,-1/2,-1/2,0),

"18. H100 - Con" = c(0,0,0,0,0,0,0,0,1/2,1/2,0,0,0,0,-1),

"19. H150 - BC" = c(0,0,0,0,0,0,0,0,0,0,1/2,1/2,-1/2,-1/2,0),

"20. H150 - Con" = c(0,0,0,0,0,0,0,0,0,0,1/2,1/2,0,0,-1))

comps12 <- glht(Model12, linfct = mcp (ID = Contrats12), alternative = "two.sided")

SC12 <- confint(comps12)

summary (SC12)

summary (comps12)

plot(SC12, main="", xlab = " Difference in Belowground Biomass (g) ")

par(mar=c (5,7,4,5)+.1)

## Washing Leaf Area

Model13 <- lm(larea~ID, data = Washing)

Contrats13 <- rbind ("1. BC - Con" = c (0,0,0,0,0,0,0,0,0,0,0,0,1/2,1/2,-1),

"2. BCM - Con" = c(0,0,0,0,0,0,0,0,0,0,0,0,1,0,-1),

"3. BCT - Con" = c(0,0,0,0,0,0,0,0,0,0,0,0,0,1,-1),

"4. BCT - BCM" = c(0,0,0,0,0,0,0,0,0,0,0,0,-1,1,0),

"5. Trt - Con" = c(1/10,1/10,1/10,1/10,0,0,1/10,1/10,1/10,1/10,1/10,1/10,0,0,-1),

"6. Trt - BC" = c(1/10,1/10,1/10,1/10,0,0,1/10,1/10,1/10,1/10,1/10,1/10,-1/2,-1/2,0),

"7. WW - BC" = c (1/4,1/4,1/4,1/4,0,0,0,0,0,0,0,0,-1/2,-1/2,0),

"8. WW - Con" = c(1/4,1/4,1/4,1/4,0,0,0,0,0,0,0,0,0,0,-1),

"9. WW24 - BC" = c(1/2,1/2,0,0,0,0,0,0,0,0,0,0,-1/2,-1/2,0),

"10. WW24 - Con" = c(1/2,1/2,0,0,0,0,0,0,0,0,0,0,0,0,-1),

"11. WW.5 - BC" = c(0,0,1/2,1/2,0,0,0,0,0,0,0,0,-1/2,-1/2,0),

"12. WW.5 - Con"= c(0,0,1/2,1/2,0,0,0,0,0,0,0,0,0,0,-1),

"13. H - Con" = c (0,0,0,0,0,0,1/6,1/6,1/6,1/6,1/6,1/6,0,0,-1),

"14. H - BC" = c (0,0,0,0,0,0,1/6,1/6,1/6,1/6,1/6,1/6,-1/2,-1/2,0),

"15. H50 - BC" = c(0,0,0,0,0,0,1/2,1/2,0,0,0,0,-1/2,-1/2,0),

"16. H50 - Con"= c(0,0,0,0,0,0,1/2,1/2,0,0,0,0,0,0,-1),

"17. H100 - BC" = c(0,0,0,0,0,0,0,0,1/2,1/2,0,0,-1/2,-1/2,0),

"18. H100 - Con" = c(0,0,0,0,0,0,0,0,1/2,1/2,0,0,0,0,-1),

"19. H150 - BC" = c(0,0,0,0,0,0,0,0,0,0,1/2,1/2,-1/2,-1/2,0),

"20. H150 - Con" = c(0,0,0,0,0,0,0,0,0,0,1/2,1/2,0,0,-1))

comps13 <- glht(Model13, linfct = mcp (ID = Contrats13), alternative = "two.sided")

SC13 <- confint(comps13)

summary (SC13)

plot(SC13, main="", xlab = " Difference in Leaf Area (cm^2) ")

par(mar=c (5,7,4,5)+.1)

##----------------------------------------------------------------------------------------------

##----------------------------------------------------------------------------------------------

## Effluents

## Aboveground biomass

Model14 <- lm(abv~ID, data = Washing)

Contrats14 <- rbind ("1. EF - Con" = c(0,0,0,0,1/2,1/2,0,0,0,0,0,0,0,0,-1),

"2. EF - BC" = c(0,0,0,0,1/2,1/2,0,0,0,0,0,0,-1/2,-1/2,0),

"3. EF24 - BC" =c(0,0,0,0,1,0,0,0,0,0,0,0,-1/2,-1/2,0),

"4. EF24 - Con"=c(0,0,0,0,1,0,0,0,0,0,0,0,0,0,-1),

"5. EF.5 - BC" =c(0,0,0,0,0,0,1,0,0,0,0,0,-1/2,-1/2,0),

"6. EF.5 - Con"=c(0,0,0,0,0,0,1,0,0,0,0,0,0,0,-1))

comps14 <- glht(Model14, linfct = mcp (ID = Contrats14), alternative = "two.sided")

SC14 <- confint(comps14)

summary (SC14)

plot(SC14, main="", xlab = " Difference in Aboveground Biomass (g) ")

par(mar=c (5,7,4,5)+.1)

## Belowground biomass

Model15 <- lm(blg~ID, data = Washing)

Contrats15 <- rbind ("1. EF - Con" = c(0,0,0,0,1/2,1/2,0,0,0,0,0,0,0,0,-1),

"2. EF - BC" = c(0,0,0,0,1/2,1/2,0,0,0,0,0,0,-1/2,-1/2,0),

"3. EF24 - BC" =c(0,0,0,0,1,0,0,0,0,0,0,0,-1/2,-1/2,0),

"4. EF24 - Con"=c(0,0,0,0,1,0,0,0,0,0,0,0,0,0,-1),

"5. EF0.5 - BC" =c(0,0,0,0,0,0,1,0,0,0,0,0,-1/2,-1/2,0),

"6. EF0.5 - Con"=c(0,0,0,0,0,0,1,0,0,0,0,0,0,0,-1))

comps15 <- glht(Model15, linfct = mcp (ID = Contrats15), alternative = "two.sided")

SC15 <- confint(comps15)

summary (SC15)

plot(SC15, main="", xlab = " Difference in Belowground Biomass (g) ")

par(mar=c (5,7,4,5)+.1)

## Leaf area

Model16 <- lm(larea~ID, data = Washing)

Contrats16 <- rbind ("1. EF - Con" = c(0,0,0,0,1/2,1/2,0,0,0,0,0,0,0,0,-1),

"2. EF - BC" = c(0,0,0,0,1/2,1/2,0,0,0,0,0,0,-1/2,-1/2,0),

"3. EF24 - BC" =c(0,0,0,0,1,0,0,0,0,0,0,0,-1/2,-1/2,0),

"4. EF24 - Con"=c(0,0,0,0,1,0,0,0,0,0,0,0,0,0,-1),

"5. EF0.5 - BC" =c(0,0,0,0,0,0,1,0,0,0,0,0,-1/2,-1/2,0),

"6. EF0.5 - Con"=c(0,0,0,0,0,0,1,0,0,0,0,0,0,0,-1))

comps16 <- glht(Model16, linfct = mcp (ID = Contrats16), alternative = "two.sided")

SC16 <- confint(comps16)

summary (SC16)

plot(SC16, main="", xlab = " Difference in Leaf Area (cm^2) ")

par(mar=c (5,7,4,5)+.1)
