# Supplementary material for: Thermal treatment and leaching of biochar alleviates plant growth inhibition from mobile organic compounds
Source: PeerJ. 2016 Aug 25;4:e2385. doi: 10.7717/peerj.2385 (PMC5012324; doi:10.7717/peerj.2385)
Supplement: Supplemental Information 5 — source code for linear regression analyses performed in experiment 1: biomass and leaf area response effect sizes as a function of increasing biochar dosage. [file peerj-04-2385-s005.docx]

Peer J- 9676

**Script for regression analysis used in Experiment 1**

* Script for contrast tests used in both experiments is available as a separate file.

## Lolium ##

doseresponse <- read.csv("~/Desktop/PeerJ (II)/Dose-response.Lolium.csv")

Exp.1MFT <- read.csv("~/Desktop/PeerJ (II)/Exp.1MFT.csv")

Exp.1MB <- read.csv("~/Desktop/PeerJ (II)/Exp.1MB.csv")

Exp.1SB <- read.csv("~/Desktop/PeerJ (II)/Exp.1SB.csv")

char <- as.factor (doseresponse$char)

## Aboveground biomass dose response Experiment 1 ##

lm1 <- lm(rr.Abv ~ Dose, data = doseresponse)

summary(lm1)

plot1 = plot (rr.Abv~Dose, col = char, data = doseresponse)+abline(lm1)

lmmft <- lm(rr.Abv ~ Dose, data = Exp.1MFT )

summary (lmmft)

plot (rr.Abv~Dose, data = Exp.1MFT)+abline(lmmft)

lmmb <- lm (rr.Abv ~ Dose, data = Exp.1MB)

summary (lmmb)

plot (rr.Abv~Dose, data = Exp.1MB)+abline(lmmb)

lmsb <- lm(rr.Abv ~ Dose, data = Exp.1SB)

summary (lmsb)

plot (rr.Abv ~ Dose, data = Exp.1SB)+abline(lmsb)

plot1 = plot (rr.Abv~Dose, pch = 16, asp=5, ylab= "Aboveground biomass (g) response ratio", xlab = "Biochar addition rate (t/ha)", col = c("black", "gray39", "dark gray"), xlim=c(5,50),frame=F, data = doseresponse)+abline(lmmft)+abline(lmmb, lty=2)+abline(lmsb, lty=3)

axis(1, at=seq(5,50,by = 5))

## Leaf area experiment 1 ##

lm2 <- lm(RR.Larea~Dose, data= doseresponse)

summary (lm2)

plot (RR.Larea~Dose, col= char, data = doseresponse)+abline(lm2)

lmmftlarea <- lm(RR.Larea~Dose, data=Exp.1MFT)

summary(lmmftlarea)

plot (RR.Larea~Dose, data = Exp.1MFT)+ abline(lmmftlarea)

lmmblarea<- lm(RR.Larea~Dose, data =Exp.1MB)

summary(lmmblarea)

plot (RR.Larea~Dose, data = Exp.1MB)+abline (lmmblarea)

lmsblarea <- lm(RR.Larea~Dose, data = Exp.1SB)

summary(lmsblarea)

plot (RR.Larea~Dose, data = Exp.1SB)+abline(lmsblarea)

plot2 = plot (RR.Larea~Dose, pch = 16, asp=5, col = c("black", "gray39", "dark gray"), xlim=c(5,50),frame=F, data = doseresponse)+abline(lmmft)+abline(lmmb, lty=2)+abline(lmsb, lty=3)

axis(1, at=seq(5,50,by = 5))

## belowground biomass experiment 1 ##

lm3 <- lm(RR.Blgb~Dose, data=doseresponse)

summary(lm3)

plot (RR.Blgb~Dose,col=char, data=doseresponse)+abline(lm3)

lmmftblgb = lm(RR.Blgb~Dose, data =Exp.1MFT)

summary(lmmftblgb)

plot(RR.Blgb~Dose, data = Exp.1MFT)+abline(lmmftblgb)

lmmbblgb=lm(RR.Blgb~Dose, data = Exp.1MB)

summary(lmmbblgb)

plot(RR.Blgb~Dose, data = Exp.1MB)+abline(lmmbblgb)

lmsbblgb = lm(RR.Blgb ~ Dose, data = Exp.1SB)

summary(lmsbblgb)

plot(RR.Blgb~Dose, data = Exp.1SB)+abline(lmsbblgb)

plot3 = plot (RR.Blgb~Dose, pch = 16, asp=5, col = c("black", "gray39", "dark gray"), xlim=c(5,50),frame=F, data = doseresponse)+abline(lmmft)+abline(lmmb, lty=2)+abline(lmsb, lty=3)

axis(1, at=seq(5,50,by = 5))

## height experiment 1 ##

lm4 <- lm(RR.height~Dose, data=doseresponse)

summary (lm4)

plot(RR.height~Dose, col = char, ylab= "response ratio height", data = doseresponse)+abline(lm4)

## Trifolium ##

trifolium <- read.csv("C:/Users/User/Desktop/PeerJ/peerj-trifolium.csv")

Exp.1MB.Tri <- read.csv("C:/Users/User/Desktop/PeerJ/Exp.1MB.Tri.csv")

Exp.1SB.Tri <- read.csv("C:/Users/User/Desktop/PeerJ/Exp.1SB.Tri.csv")

Exp.1MFT.Tri <- read.csv("C:/Users/User/Desktop/PeerJ/Exp.1MFT.Tri.csv")

View (trifolium)

## aboveground biomass ##

lm5 <- lm (rr.abvg ~ Dose, data = trifolium)

summary (lm5)

plot (rr.abvg ~ Dose, col = Biochar, data = trifolium)+ abline (lm5)

lmmftabvg<-lm(rr.abvg~Dose, data = Exp.1MFT.Tri)

summary(lmmftabvg)

plot (rr.abvg~Dose, data =Exp.1MFT.Tri)+abline(lmmftabvg)

lmmbabvg <-lm(rr.abvg~Dose, data = Exp.1MB.Tri)

summary(lmmbabvg)

plot (rr.abvg~Dose, data= Exp.1MB.Tri)+abline(lmmbabvg)

lmsbabvg <- lm(rr.abvg~Dose, data = Exp.1SB.Tri)

summary(lmsbabvg)

plot (rr.abvg~Dose, data=Exp.1SB.Tri)+abline(lmsbabvg)

plot (rr.abvg ~ Dose, col = Biochar, data = trifolium)+ abline (lmmftabvg)+abline(lmmbabvg)+abline(lmsbabvg)

## belowground biomass ##

lm6 <- lm (rr.blg ~ Dose, data = trifolium)

summary (lm6)

plot (rr.blg ~ Dose, col = Biochar, data = trifolium)+abline (lm6)

lmmftblg <- lm (rr.blg~Dose, data = Exp.1MFT.Tri)

summary(lmmftblg)

plot (rr.blg ~ Dose, data = Exp.1MFT.Tri) + abline (lmmftblg)

lmmbblg <- lm (rr.blg~Dose, data = Exp.1MB.Tri)

summary (lmmbblg)

plot (rr.blg~Dose, data = Exp.1MB.Tri)+abline(lmmbblg)

lmsbblg <-lm (rr.blg~Dose, data = Exp.1SB.Tri)

summary(lmsbblg)

plot (rr.blg~Dose, data = Exp.1SB.Tri)+abline(lmsbblg)

## nodules ##

lm7 <- lm (rr.nods~Dose, data = trifolium)

summary (lm7)

plot (rr.nods~Dose, col = Biochar, data = trifolium)+abline (lm7)

lmmftnods <- lm (rr.nods~Dose, data = Exp.1MFT.Tri)

summary(lmmftnods)

plot (rr.nods ~ Dose, data = Exp.1MFT.Tri) + abline (lmmftnods)

lmmbnods <- lm (rr.nods~Dose, data = Exp.1MB.Tri)

summary (lmmbnods)

plot (rr.nods~Dose, data = Exp.1MB.Tri)+abline(lmmbnods)

lmsbnods <-lm (rr.nods~Dose, data = Exp.1SB.Tri)

summary(lmsbnods)

plot (rr.nods~Dose, data = Exp.1SB.Tri)+abline(lmsbnods)
